# Precision patient education using a “flipped classroom” approach

**DOI:** 10.1002/acm2.13601

**Published:** 2022-04-28

**Authors:** Bradley W Schuller, Christina Burch, Theresa Casterton, Catie Crowther, Jordan Fowler, Matthew H Stenmark

**Affiliations:** ^1^ SCL Health, St. Joseph Hospital Radiation Oncology Denver Colorado USA; ^2^ SCL Health Broomfield Colorado USA; ^3^ Kaiser Permanente, St. Joseph Hospital Radiation Oncology Denver Colorado USA

**Keywords:** active learning, flipped classrooms, patient education, telemedicine

## Abstract

**Objectives:**

To improve patient education delivered over telemedicine by using a “flipped classroom”‐inspired approach.

**Methods:**

A “flipped classroom” is an education strategy used to engage active learning by sending students home with lecture material and reserving classroom time for collaborative learning. To adapt this approach for use in radiation oncology patient education, three pieces of written education material were created: introduction to radiation oncology, treatment planning scan, and treatment delivery. An automated system was created to deliver precisely timed emails at three time points ahead of appointments. Appointment time was then used for collaborative learning with our staff. As a primary endpoint, email engagement metrics were tracked via the automated system. Secondarily, enrolled patients were surveyed to assess level of understanding (before vs. after intervention), anxiety (before vs. after intervention), and satisfaction. Additionally, email delivery timing, clarity, relevance, and patient support were evaluated. Data analyses test the impact of active learning against our existing education approaches.

**Results:**

Overall, 77.1% of the emails were opened, and of those, patients accessed 72.2% of the education material. Patients re‐read the education material 4.6 times on average. Active learning increased patient understanding regarding the purpose of the treatment planning scan (*p* = 0.031) and increased patient understanding of what to expect during daily radiation treatments (*p* = 0.0078). Patients reported reduced anxiety (*p* = 0.031) and high scores for satisfaction, timing, clarity, relevance, and overall support.

**Conclusions:**

Patient engagement with the education material was high, and they continued to access it many times. Active learning enhances patient comprehension of complex treatment information leading to decreased anxiety. Furthermore, this technique can be incorporated into existing telemedicine with basic technology.

## 1 INTRODUCTION

Telemedicine usage in oncology has undergone recent expansion.^1^ However, virtual patient interaction is difficult to manage, especially for appointments that require teaching and high volumes of information transfer between clinician and patient.^1^ Effective patient education requires teaching at every point along the patient's care path, which is difficult in a traditional clinical medicine setting. Advances in the science of learning espouse the benefits of active learning to enhance memory formation and increase student performance.^2^, ^3^ A “flipped classroom” is an educational approach designed to engage active learning by transferring much of the didactic component traditionally reserved for the classroom to the student's home, leaving classroom time for collaborative learning with teachers and peers.^4^ “Core features of the flipped learning approach include: content in advance (generally a pre‐recorded lecture), educator awareness of students’ understanding, and higher order learning during class time.^4^” Telemedicine provides a platform to adopt active learning by enabling a “flipped classroom” approach to patient education, where the technique's core features are easily implemented.

In the radiation oncology clinic, patients typically gather health information from a myriad of different sources, which include direct consultation with radiation oncologists, mid‐level providers, nursing staff, and radiation therapists. This is typically augmented by internet research and anecdotal advice from family and friends. However, recent studies have shown that many online information sources are inaccurate^7^ or too complex.^8^, ^9^, ^10^ A “flipped classroom” approach to patient education delivers tailored information to patients at the right time, which may reduce their need to explore information sources beyond the control of the treating department. This information can be department specific, which creates a direct connection to the actual experience in the department during treatment.

For this study, we developed an automated system to send patients relevant information at precisely timed points ahead of future appointments to engage active learning. Here, we report the findings of a pilot study designed to evaluate the early benefits of this approach for our patients.

## 2 METHODS

Our institution's IRB determined that the project did not meet the definition of research and designated it quality improvement. The study was conducted between June 1 and August 31, 2020 and focused on a subsection of the radiation oncology care path between the initial physician consult, treatment planning scan, and first treatment day. Data show that most patient requests for additional information pertain to this part of the care path.^5^ Specifically, the Geinitz study reported that most patient questions pertained to radiation delivery and radiation side effects: “How do x‐rays carry out their effect?” (65.2% of requests), “Function of the linear accelerator” (59.8%), and “What are x‐rays?” (52.1%). These data are further supported by recent publications from the Atwood et al. studies evaluating trends in patient questions during physicist‐directed patient consults^14^ and data from our own group.^15^

Three pieces of written education material were created: (A) introduction to radiation oncology, (B) treatment planning scan, and (C) treatment delivery. Each piece consisted of 1.5–2 pages of text written at an appropriate reading level as recommended by numerous medical organizations, including the American Medical Association and National Institutes of Health. Material A explained why radiation is used to treat cancer, defined some common radiation oncology terms, and introduced the entire care team. Material B explained the basic concepts behind CT scans, tissue segmentation, planning approaches, and dose calculations. Material C presented a general overview of linear accelerator operation and the basics of what to expect in the treatment room on the first day of treatment. The text was further augmented using graphics and illustration to clarify some of the more difficult concepts. Each piece was customized for disease site, not individual patients. As an example, similar approaches to developing patient education materials have been used in the ASTRO patient education brochures.^16^

Our institution's customer relationship management (CRM) software (Salesforce, San Francisco, CA) was programmed to monitor a patient's treatment schedule, adapt dynamically to schedule changes if needed, and deliver precisely timed emails at three time points: (1): immediately following the physician consult (material (A) delivered); (2): 24 hrs after email (1) with as much time as possible before the treatment planning scan (material (B) delivered); and (3): 2 days before the first treatment day (material (C) delivered). We chose these time points based on the concentration of patient questions around this part of the patient care path as described previously. This decision was further enforced by our team discussions during study planning, which identified weaknesses in our existing patient education program during these time points.

Since material was delivered ahead of time, appointments were used for collaborative learning. The appointments were not modified from their original intent or timing. For example, a study patient's treatment planning scan appointment was kept the same in terms of scheduling and overall intent. Rather, because patients had education material ahead of each appointment, the appointment content and discussion changed from largely didactic lecturing to question‐and‐answer‐ and clarification‐based discussions. Each appointment was managed by the typical staff involved, which consisted largely of physician, therapist, and physicist interactions.

Patients with breast and prostate cancer were enrolled. As the primary endpoint, email engagement metrics were tracked via the CRM software. Specifically, email open rates, education material open rates, average material read times, and average number of times the education material was accessed were evaluated. Following the completion of the education program, enrolled patients were surveyed to assess the level of understanding (before vs. after intervention), anxiety (before vs. after intervention), and satisfaction. Survey responses regarding the level of understanding were self‐assessments of understanding. Additionally, email delivery timing, clarity, relevance, and patient support were evaluated.

Data analyses test the impact of active learning against our existing education approaches (i.e., “Before intervention” is the patient's education status resulting from our existing education program, while “After intervention” is the patient's education status following the additional “flipped classroom” approach). Survey questions were adapted from validated sources and used a five‐point Likert scale (Supporting Information).^12^ Comparison statistics between before and after survey points were generated using a Wilcoxon signed‐rank test for matched pairs (one‐tailed [a priori prediction of a unidirectional effect]; *α* = 0.05; GraphPad Prism 8, San Diego, CA).

## 3 RESULTS

In total, 26 patients were enrolled (9/17 [no. of breast/no. of prostate]). The survey response rate was 48% (Table 1). One survey response was removed due to lack of participation. Due to technical errors, two patients never received emails, and four patients never received email no. 3.

**TABLE 1:** Program engagement metrics

Enrolled patients	26
Breast/prostate split	9/17 (35%/65%)
**Email engagement**	
No. who opened 0 emails	3
No. who opened one email	1
No. who opened two emails	7
No. who opened three emails	13
No. engaged with at least one email	21
Survey response rate % (no. of survey responses/no. of engaged patients)	48% (10/21)
**Email open rate % (no. who opened emails/no. of emails delivered)**	
Time point (1): introduction to radiation oncology	87.5% (21/24)
Time point (2): treatment planning scan	70.8% (17/24)
Time point (3): treatment delivery	72.7% (16/22)
Overall email open rate	77.1% (54/70)
**Education material open rate % (no. who opened material/no. who opened emails)**	
Time point (1): introduction to radiation oncology	61.9% (13/21)
Time point (2): treatment planning scan	70.6% (12/17)
Time Point (3): treatment delivery	87.5% (14/16)
Overall education material open rate	72.2% (39/54)
**Avg. no. of times material accessed per patient (no. of times accessed/no. who opened material)**	
Time point (1): introduction to radiation oncology	4.4 (57/13)
Time point (2): treatment planning scan	5.2 (62/12)
Time point (3): treatment delivery	4.4 (61/14)
**Avg. material read time**	
Time point (1): introduction to radiation oncology	3.0 min
Time point (2): treatment planning scan	1.6 min
Time point (3): treatment delivery	2.2 min

Email open rates were 21/24 (no. who opened emails/no. of emails delivered), 17/24, and 16/22 for delivery time points 1, 2, and 3, respectively. Of the patients who opened the emails, 13/21 (no. who opened material/no. who opened emails), 12/17, and 14/16 engaged with the education material during delivery time points 1, 2, and 3, respectively. Patients accessed and re‐read the education material 4.6 times on average (Table 1).

**FIGURE 1:**
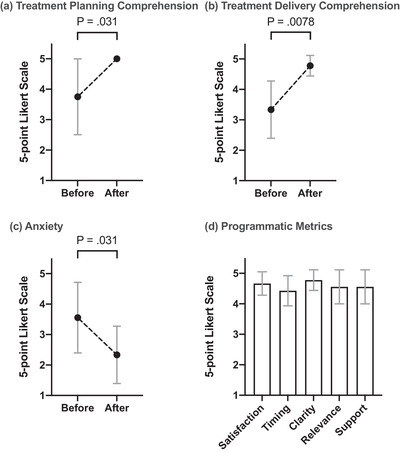
(a) I understood the purpose of my treatment planning scan (1 “Disagree” vs. 5 “Agree”). (b) I knew what to expect for my daily radiation treatments (1 “Disagree” vs. 5 “Agree”). (c) My anxiety level (1 “Low” vs. 5 “High”). (d) Programmatic metrics for patient satisfaction, information delivery timing, information clarity, information relevance, and patient support. Error bars represent 95% CI

Active learning increased patient understanding regarding the purpose of the treatment planning scan (before vs. after intervention [Figure 1a]: Avg, 3.7; 95% CI, 2.5–4.9 vs. Avg, 5.0; 95% CI, 5.0–5.0; *p* = 0.031) and increased patient understanding of what to expect during daily radiation treatments (before vs. after intervention [Figure 1b]: Avg, 3.3; 95% CI, 2.4–4.3 vs. Avg, 4.8; 95% CI, 4.4–5.1; *p* = 0.0078). Patients reported reduced anxiety (before vs. after intervention [Figure 1c]: Avg, 3.6; 95% CI, 2.4–4.7 vs. Avg, 2.3; 95% CI, 1.4–3.3; *p* = 0.031) and high scores for satisfaction, timing, clarity, relevance, and overall support (Figure 1d).

## 4 DISCUSSION

This study demonstrates that active learning increases patient understanding of difficult treatment information and can be effectively incorporated into existing telemedicine efforts using basic technology. Patient engagement with the education material was high, especially for a subset of engaged patients, and they continued to access and re‐read the material many times. These observations support the foundational basis that “flipped classroom” approaches give recipients the opportunity to process information at their own pace.^4^

Data comparisons against existing telemedicine benchmarks show that our education program engagement rates far exceeded typical telemedicine engagement metrics. Lin et al. showed that although 95% of patients were given access to their electronic health records, only 10% actually used it.^13^ Even though our targeted education material may not formally be regarded as being part of the formal electronic health record, the Lin study nevertheless showed that electronic access does not necessarily translate to true patient engagement. Our study was able to show high patient engagement despite the electronic formatting.

Technology facilitates active learning.^4^ Many institutions have been successful delivering education content over the internet (e.g., MITx, edX, and Khan Academy). In a similar fashion, targeted delivery of health information is possible with modern electronic medical record systems seen in most healthcare institutions. This infrastructure is already commonly used to transmit personal medical records to patients. Our data show that patients readily engage with medical information delivered electronically. Overall, 77.1% (54/70) of the emails were opened, and of those, 72.2% (39/54) of the education information was opened. Patients accessed the information more than four times on average and spent a considerable amount of time reading the information. The high scores patients reported for material clarity indicate that patients were engaging in active learning and not accessing the materials multiple times because of confusion or comprehension problems.

New strategies for effective patient education are needed to enhance information transfer between patients and clinicians in a time of increased telemedicine usage. Given appointment time constraints, most of the time may be spent discussing the immediate medical condition, leaving very little time to discuss other concerns.^6^ Our “flipped classroom” approach to patient education solves this problem by creating space for collaborative learning during appointment time, as many of the routine didactic components were transferred to the patient's home. This gives patients time to digest the information and formulate questions.

Recent studies have shown that elevated levels of patient distress can negatively impact long‐term survival.^11^ Although patient anxiety is difficult to assess, our results are promising that active learning strategies may have an anxiety‐reducing effect. Further study is required to establish a definitive answer. Technical patient education via in‐person medical physics consults has been shown to have an anxiety‐reducing effect.^12^ Novel strategies for patient education will likely become increasingly important with future advances in healthcare delivery.

Overall, our study is limited by its small cohort size, but the engagement data are compelling and warrant further study either by expanding the breadth of education offerings or increasing enrollment numbers. Potential bias may be present in the dataset as patients were asked to self‐assess their understanding of the materials and reflect on a state of being prior to receiving the flipped classroom intervention. Another limitation is that the study was not a controlled trial. The data analyses test the effectiveness of the “flipped classroom” intervention against our existing education practice and do not control for additional external education provided to, or accessed by, the patient. The study also presents data that were derived from a single cohort of patients. This pilot study was designed to probe for effects that could be used to inform a more rigorous study design to assess the “flipped classroom” approach. Future studies will utilize a randomized controlled trial with a larger sample size.

## CONFLICT OF INTEREST

No conflicts of interest.

## FUNDING INFORMATION

SCL Health Innovation Challenge Grant

## AUTHOR CONTRIBUTION

Bradley W Schuller: conceptualization, methodology, validation, formal analysis, investigation, data curation, writing—original draft, writing—review and editing, visualization, supervision, project administration, funding acquisition

Christina Burch: methodology, investigation, resources, writing—review and editing

Theresa Casterton: conceptualization, methodology, resources, writing—review and editing, supervision

Catie Crowther: methodology, software, formal analysis, resources, data curation, writing—review and editing

Jordan Fowler: methodology, investigation, resources, writing—review and editing

Matthew H Stenmark: formal analysis, writing—review and editing

## Supporting information

Supporting Information
