# Supplementary material for: Precision patient education using a “flipped classroom” approach
Source: J Appl Clin Med Phys. 2022 Apr 28;23(5):e13601. doi: 10.1002/acm2.13601 (PMC9121034; doi:10.1002/acm2.13601)
Supplement: Supplementary file 1 — Supporting Information [file ACM2-23-e13601-s001.docx]

**Supplement**

Section 1.

Before receiving the education emails, I understood the purpose of my treatment planning scan.

Disagree 1 2 3 4 5 Agree

After receiving the education emails, I better understood the purpose of my treatment planning scan.

Disagree 1 2 3 4 5 Agree

Section 2.

Before receiving the education emails, I knew what to expect for my daily radiation treatments.

Disagree 1 2 3 4 5 Agree

After receiving the education emails, I knew what to expect for my daily radiation treatments more clearly.

Disagree 1 2 3 4 5 Agree

Section 3.

My anxiety level before starting treatment:

Low 1 2 3 4 5 High

My anxiety level after receiving the education emails:

Low 1 2 3 4 5 High

Section 4.

How much did the education emails contribute to your overall satisfaction with your care?

Not at all 1 2 3 4 5 Very much

The education emails were delivered at the right moment during my care.

Disagree 1 2 3 4 5 Agree

The education information was presented clearly.

Disagree 1 2 3 4 5 Agree

The education emails were pertinent to my care.

Disagree 1 2 3 4 5 Agree

I had enough opportunity to ask questions.

Disagree 1 2 3 4 5 Agree
